# Detection of Increased Plasma Interleukin-6 Levels and Prevalence of *Prevotella copri* and *Bacteroides vulgatus* in the Feces of Type 2 Diabetes Patients

**DOI:** 10.3389/fimmu.2017.01107

**Published:** 2017-09-15

**Authors:** Aline Zazeri Leite, Nathália de Campos Rodrigues, Marina Ignácio Gonzaga, João Carlos Cicogna Paiolo, Carolina Arantes de Souza, Nadine Aparecida Vicentini Stefanutto, Wellington Pine Omori, Daniel Guariz Pinheiro, João Luiz Brisotti, Euclides Matheucci Junior, Vânia Sammartino Mariano, Gislane Lelis Vilela de Oliveira

**Affiliations:** ^1^Microbiome Study Group, School of Health Sciences Dr. Paulo Prata (FACISB), Barretos, Brazil; ^2^QGene-Solutions and Logistics in Health, Sao Carlos, Brazil; ^3^Board of Health from Barretos, Barretos, Brazil; ^4^Department of Technology, School of Agricultural and Veterinarian Sciences, São Paulo State University (UNESP), Sao Paulo, Brazil; ^5^DNA Consult Genetics and Biotechnology, Sao Carlos, Brazil; ^6^Biotechnology Department, Sao Carlos Federal University, UFSCAR, Sao Carlos, Brazil; ^7^Barretos Cancer Hospital (HCB), Barretos, Brazil

**Keywords:** type 2 diabetes, dietary habits, intestinal microbiota, inflammatory cytokines, interleukin-6, metabolic endotoxemia

## Abstract

Intestinal dysbiosis and metabolic endotoxemia have been associated with metabolic disorders, such as obesity, insulin resistance, and type 2 diabetes (T2D). The main goal of the present study was to evaluate the intestinal dysbiosis in Brazilian T2D patients and correlate these data with inflammatory cytokines and lipopolysaccharides (LPS) plasma concentrations. This study was approved by the Ethics Committees from Barretos Cancer Hospital and all individuals signed the informed consent form. Stool samples were required for DNA extraction, and the V3/V4 regions of bacterial 16S were sequenced using an Illumina platform. Peripheral blood was used to quantify inflammatory cytokines and plasma LPS concentrations, by CBA flex and ELISA, respectively. Statistical analyses were performed using Mann–Whitney and Spearman’s tests. Analysis of variance, diversity indexes, and analysis of alpha- and beta-diversity were conducted using an annotated Operational Taxonomic Unit table. This study included 20 patients and 22 controls. We observed significant differences (*P* < 0.01) in the microbiota composition (beta-diversity) between patients and controls, suggesting intestinal dysbiosis in Brazilian T2D patients. The prevalent species found in patients’ feces were the Gram-negatives *Prevotella copri, Bacteroides vulgatus, Bacteroides rodentium*, and *Bacteroides xylanisolvens*. The proinflammatory interleukin-6 (IL-6) was significantly increased (*P* < 0.05) in patients’ plasma and LPS levels were decreased. We find correlations between the proinflammatory interferon-gamma with Gram-negatives *Bacteroides* and *Prevotella* species, and a positive correlation between the LPS levels and *P. copri* reads. The *P. copri* and *B. vulgatus* species were associated with insulin resistance in previous studies. In this study, we suggested that the prevalence of Gram-negative species in the gut and the increased plasma IL-6 in patients could be linked to low-grade inflammation and insulin resistance. In conclusion, the *P. copri* and *B. vulgatus* species could represent an intestinal microbiota signature, associated with T2D development. Furthermore, the identification of these Gram-negative bacteria, and the detection of inflammatory markers, such as increased IL-6, could be used as diabetes predictive markers in overweight, obese and in genetically predisposed individuals to develop T2D.

## Introduction

Type 2 diabetes (T2D) is a chronic disease characterized by insulin resistance, glucose intolerance, fat deposition, dyslipidemia, and systemic inflammation (1). According to the International Diabetes Federation, diabetes will affect 642 million people worldwide until 2040 (2). T2D development involves genetic and environmental factors, and recent reports have implicated the gut microbiota in the regulation of glucose and lipid metabolism (3, 4). Consistently, some studies correlate important perturbations in the gut microbiota composition with systemic inflammation, which is observed in metabolic dysfunctions, such as insulin resistance, obesity, and T2D (3–5).

In humans, more than a trillion of microorganisms, primarily bacteria, colonize the oral-gastrointestinal tract and reside in the distal portion of the intestine (6, 7). The gut microbiota contributes to many of the host physiological processes, and in turn, the host offers niche and nutrients for the survival of these microbes (8–10). The main contributions of the gut microbiota include digestion and carbohydrate fermentation, vitamins synthesis, mucosal lymphoid tissue development, epithelial barrier maintenance, and the prevention of pathobionts colonization (11–13). Furthermore, the interaction between host immune system and gut microbiota is necessary for the maintenance of mucosal immune homeostasis and epithelial barrier integrity (14). However, the interruption of this healthy interaction, with an imbalance in the normal bacterial ecology in the gut, defined as intestinal dysbiosis, may contribute to the development of metabolic and chronic inflammatory diseases, such as T2D (14–16).

Some mechanisms have been proposed to explain the influence of the gut microbiota on insulin resistance and T2D, such as bacterial translocation, metabolic endotoxemia, defective secretion of incretins, and decreased butyrate concentrations (13, 17). We focused on metabolic endotoxemia, involving the release of proinflammatory cytokines, such as tumor necrosis factor (TNF), interleukin-1 (IL-1), and interleukin-6 (IL-6), in response to the activation of innate immune receptors by lipopolysaccharides (LPS). LPS are endotoxins present in the cell wall of Gram-negative bacteria, and these molecules are primarily responsible for the endotoxemia observed in metabolic disorders (18).

Changes in Gram-negative bacteria concentrations in the gut, combined with increased intestinal permeability, could promote LPS escape into the bloodstream and induce systemic inflammation (19). Inflammation is associated with insulin resistance because the proinflammatory cytokines block insulin signaling by inhibiting the phosphorylation of insulin receptors (20, 21). Furthermore, studies in animal models showed that high-fat diets promote an increase in Gram-negative bacteria in the gut and increased LPS absorption in the intestinal mucosa (21). In addition, induction of metabolic endotoxemia *via* intravenous administration of LPS in mice induces a fast increase in glycemia, insulinemia, and dyslipidemia (21, 22).

Recent reports have suggested that increased IL-6 plasma concentrations, C-reactive proteins, and intestinal dysbiosis are associated with obesity and T2D development (23–26). Some studies showed that proinflammatory bacteria, *Ruminococcus gnavus* and *Bacteroides* spp., are more prevalent in the feces of T2D patients (25, 26). However, butyrate-producing bacteria, such as *Roseburia intestinalis* and *Faecalibacterium prausnitzii*, with anti-inflammatory effects, were decreased in T2D patients (25).

Based on studies showing the importance of resilience of the intestinal microbiota in human health and studies showing that intestinal dysbiosis may be strongly associated with gut epithelial barrier disruption, bacterial translocation, and metabolic endotoxemia (3–5, 27), we hypothesized that the abundance of Gram-negative bacteria is greater in the feces of T2D patients and positively correlated with the plasma levels of proinflammatory cytokines and LPS. In the present study, we evaluated intestinal dysbiosis in Brazilian T2D patients and correlated these data with systemic inflammatory cytokines and plasma LPS concentrations.

## Materials and Methods

### Patients and Controls Enrollment

Type 2 diabetes patients with fasting blood glucose levels greater than or equal to 126 mg/dL (2) were enrolled at the Board of Health from Barretos, Sao Paulo, Brazil, from June 1st, 2015 to July 30th, 2016. A physician from the endocrinology department selected a total of 20 patients, 11 females and 9 males, ranging from 36 to 75 years (mean age ± SD = 58.9 ± 8.4 years) for inclusion in the present study.

Healthy controls, without T2D familial history among grandparents, parents, and siblings, were enrolled. A total of 22 healthy controls, 12 males and 10 females, ranging from 36 to 69 years (mean age ± SD = 55.7 ± 8.3 years) were enrolled in the present study. The present study was performed in accordance with the recommendations of Ethics Committee from Barretos Cancer Hospital. All subjects provided written informed consent in accordance with the Declaration of Helsinki. The protocol was approved by the Barretos Cancer Hospital (Process number 903/2014). Subsequent to consent, the peripheral blood of patients and controls was collected and stool samples were requested, and its delivery occurred within 5 days.

All subjects who had used anti-inflammatories, antibiotics, and laxatives in the last 15 days prior to blood and feces collection were excluded in the present study. Similarly, all individuals who were vaccinated or administered corticosteroids in the last 30 days were not included. The presence of chronic diarrhea and surgeries, such as appendectomy, cholecystectomy, and bariatric surgery, were also considered as exclusion criteria for T2D patients and healthy controls.

Clinical data from T2D patients, such as body mass index (BMI), fasting blood glucose (close to blood collection), glycated hemoglobin (HbA1_C_), and disease duration, were recorded. Table 1 summarizes the demographic and clinical data from T2D patients.

**Table 1:** Demographic and clinical features obtained from 20 type 2 diabetes (T2D) patients.

Patients	Gender/age	Ethnicity	BMI (kg/m^2^)	Fasting blood glucose (mg/dL)	HbA1_C_ (%)	Disease duration (years)
T2D01	M/49	Caucasian	27.8	191.0	8.0	16
T2D02	M/58	Caucasian	40.1	255.1	8.6	10
T2D03	F/62	Caucasian	32.4	87.0	7.4	11
T2D04	M/63	Caucasian	27.8	100.0	9.8	20
T2D05	M/64	Asian	32.0	76.0	5.3	16
T2D06	F/61	Afrodescendent	37.8	175.3	8.2	10
T2D07	M/58	Afrodescendent	27.5	98.6	9.8	10
T2D08	M/68	Afrodescendent	30.1	ND	ND	1
T2D09	F/36	Caucasian	30.5	84.3	6.8	9
T2D10	F/61	Caucasian	29.1	271.0	8.0	5
T2D11	M/63	Caucasian	27.4	96.0	9.9	21
T2D12	F/66	Caucasian	37.8	258.0	9.2	9
T2D13	F/45	Caucasian	31.2	245.0	7.7	18
T2D14	F/54	Caucasian	26.0	174.0	8.0	8
T2D15	M/59	Afrodescendent	31.6	ND	ND	ND
T2D16	F/75	Caucasian	21.1	220.0	8.5	21
T2D17	M/60	Caucasian	21.8	ND	ND	6
T2D18	F/56	Caucasian	27.7	ND	ND	17
T2D19	F/63	Caucasian	28.0	ND	ND	1
T2D20	F/57	Caucasian	35.6	ND	ND	ND

*M, male; F, female; BMI, body mass index (BMI > 25 = overweighted, BMI > 30 = obese); HbA1_C_, glycated hemoglobin (reference: 4.0–6.0%); ND, not determined. Fasting blood glucose (reference < 126 mg/dL)*.

### Bacterial DNA Extraction, V3/V4 Amplification, and Sequencing

Bacterial DNA was extracted from 250 mg of stool using a PowerSoil DNA Isolation Kit (MO BIO Laboratories, QIAGEN, CA, USA), according to the manufacturer’s instructions. The amount of DNA was determined using a Quantus fluorometer and adjusted to 5 ng/mL with Tris buffer (10 mM, pH 8.5). The V3 and V4 regions of the bacterial 16S were amplified by PCR using 2.5 mL of bacterial DNA, 5 mL of each primer and 12.5 mL of 2× KAPA HiFi HotStart Ready Mix (Kapa Biosystems, MA, USA). The PCR products were purified using an XP AMPure Beads Kit (BD Biosciences, CA, USA). DNA libraries were constructed according to the Illumina protocols, including steps of connecting adapters (Nextera XT Index Kit), purification steps (AMPure XP beads), quantification, and pre-denaturation (V3 MiSeq reagent kits). Sample sequencing was performed using an Illumina MiSeq platform system.

### Cytokine Quantification Using a Cytometric Bead Array

Peripheral blood (10 mL) was collected from T2D patients and controls and plasma-EDTA was separated by centrifugation at 1,372 *g*, for 10 min, 4°C. Cytokine quantification was performed by cytometric bead array (Human Th1/Th2/Th17 Cytokine Kit, BD Biosciences, CA, USA). Plasma levels of IL-2, IL-4, IL-6, IL-10, IL-17A, TNF, and interferon-gamma (IFN-γ) were detected by flow cytometer FACSCanto™ II (BD Biosciences). The analyses were performed by using BDFCAP array™ software and results were expressed by conversion of the median fluorescence intensity in picograms per milliliter.

### LPS Quantification by ELISA Assay

After centrifuging the blood samples, plasma-EDTA was stored at −80°C until the measurement of LPS. Plasma samples were used for LPS quantification, performed in duplicate, using a LPS ELISA Kit (LPS, Elabscience Biotechnology, MD, USA), according to the manufacturer’s instructions. The absorbance was read at 450 nm, and the results are presented as nanograms per milliliter.

### Statistical Analyses and Paired-End Data Processing

The comparisons between cytokines and LPS levels in patients and controls were performed using the Mann–Whitney test (28). The correlations among the relative abundance of microbiota with cytokines, LPS levels, and clinical data were performed using the Spearman’s test (29). *P* values less than 0.05 were considered statistically significant.

The paired-end reads from sequenced 16S amplified fragments were first assembled using PANDAseq v.2.10 (30) and subsequently processed using Cutadapt v.1.12 (31) to trim the Illumina adapter sequences, and PRINSEQ v.0.19.5 (32) to evaluate reads quality and to trim off low-quality bases from 3′ end from reads. Thus, we only retrieved high quality sequences between 350 and 500 bases in size to identify the Operational Taxonomic Units (OTUs) associated with each library. Chimeric sequence analyses, clustering, taxonomic assignment, and statistical analysis were performed using QIIME v.1.9.1 scripts (33), according to Souza and colleagues (34), with minor modifications, as described as follows. To obtain an OTU table, we first performed a multiple sequence alignment with MUSCLE v.3.8.31 (35), together with the pre-aligned 16S data from SILVA 119 database. Chimeric sequences were identified and removed and clustering was performed using the UPARSE protocol (36). The taxonomic assignment for each OTU was based on Ribosomal Database Project (RDP-II) (37) trainset 14 using MOTHUR v.1.25 (38). Analysis of variance, diversity index (Shannon and Observed species), and analysis of α- and β-diversity were conducted from the annotated OTU table.

## Results

### Prevalence of Gram-Negative Species in the Feces of T2D Patients

**Figure 1:**
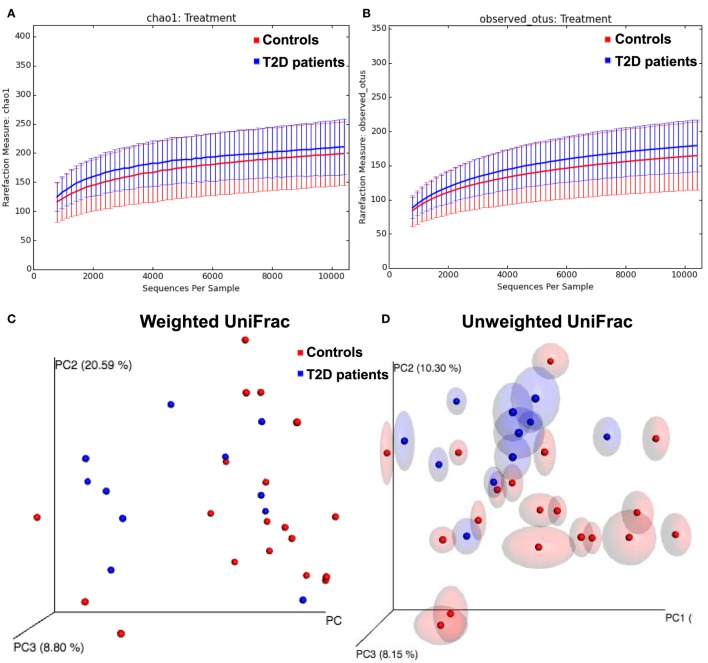
Alpha and beta-diversity in gut microbiota from type 2 diabetes (T2D) patients. **(A,B)** Rarefaction curves comparing the species richness, Chao1, and observed Operational Taxonomic Unit (OTU) numbers. **(C,D)** PcoA plots with weighted and unweighted UniFrac metric with Bonferroni’s correction.

To evaluate the intestinal dysbiosis in T2D patients, we analyzed the 16S ribosomal DNA sequences in stool samples obtained from patients and controls, targeting V3–V4 conserved regions. We calculated alpha and beta-diversity to evaluate differences in microbiota community in patients and healthy subjects. There were no significant differences (*P* < 0.05) in richness (Chao1 and observed OTUs) and evenness (Shannon and Simpson) measured according to the rarefaction curves between T2D patients and controls (Table 2; Figures 1A,B). However, using the weighted and unweighted UniFrac metric with Bonferroni’s correction (beta-diversity), we observed that the microbial communities from T2D patients and healthy controls were not similar (*P* = 0.01) (Figures 1C,D).

**Table 2:** Diversity and richness index results from alpha-diversity analyses.

Sample ID	Simpson	Shannon	Chao1	Observed Operational Taxonomic Units
T2D01	0.95	5.53	240.44	213
T2D04	0.95	5.26	194.55	167
T2D07	0.92	5.20	267.12	231
T2D08	0.91	4.46	107.00	99
T2D09	0.92	4.90	196.16	165
T2D11	0.92	5.00	219.05	182
T2D13	0.89	4.47	149.17	138
T2D14	0.93	4.98	215.15	180
T2D15	0.96	5.67	257.02	227
T2D17	0.92	5.17	272.06	209
CTL16	0.94	5.22	164.86	148
CTL22	0.94	4.90	172.76	127
CTL23	0.93	5.28	221.05	184
CTL29	0.95	5.36	205.35	178
CTL31	0.96	5.88	286.21	224
CTL32	0.93	4.56	108.50	83
CTL33	0.97	6.11	284.67	264
CTL34	0.91	4.78	200.62	159
CTL37	0.90	4.23	164.25	141
CTL41	0.89	4.47	197.60	148
CTL43	0.86	4.05	115.10	98
CTL44	0.94	5.02	138.50	110
CTL45	0.93	5.21	229.91	196
CTL46	0.90	4.22	132.00	94
CTL47	0.96	5.67	274.22	227
CTL49	0.97	5.94	262.88	231
CTL50	0.94	5.28	245.13	204
CTL51	0.91	4.64	161.00	143

**Figure 2:**
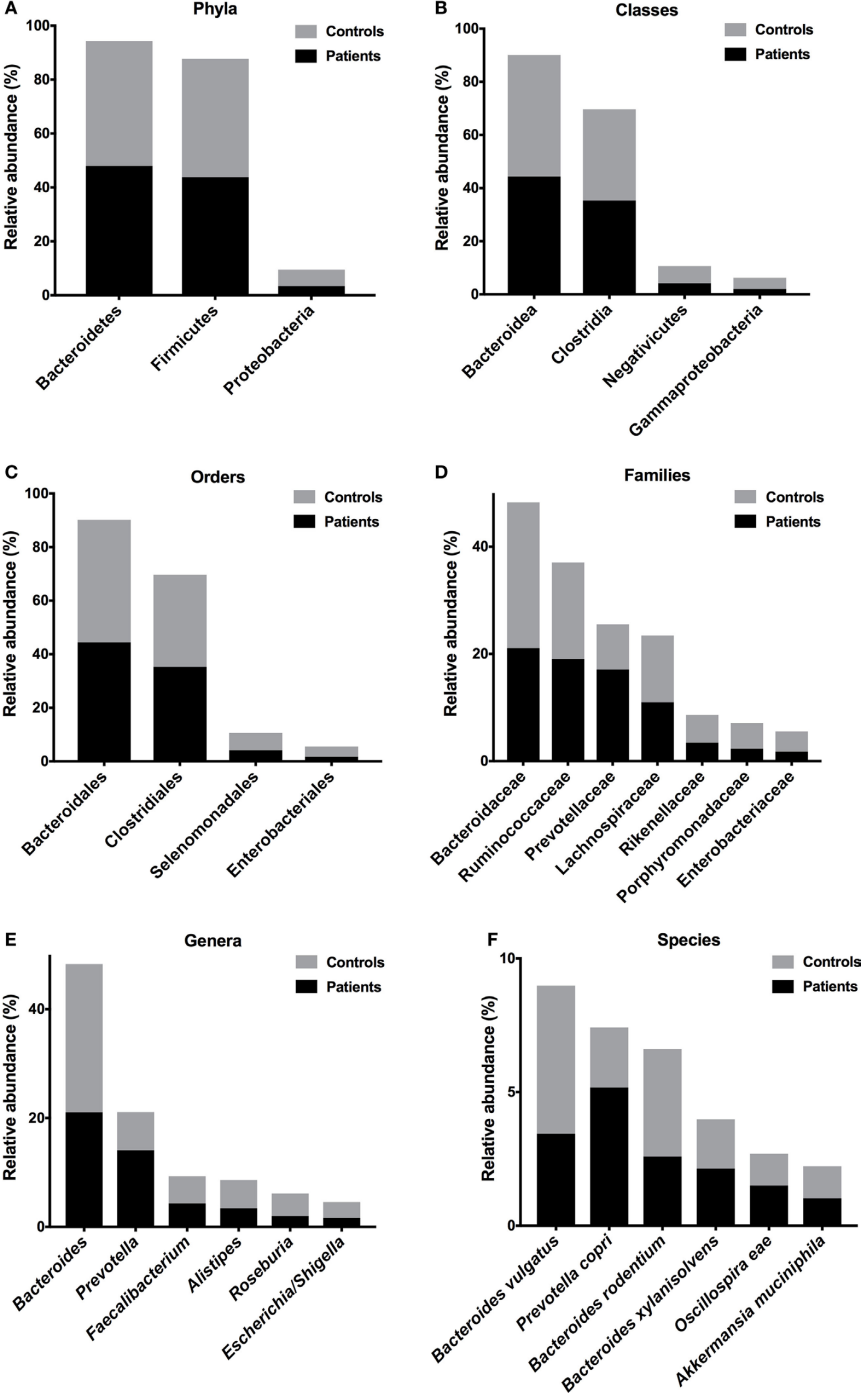
Relative abundances of bacterial taxa in the feces of T2D patients. Prevalent phyla **(A)**, classes **(B)**, orders **(C)**, families **(D)**, genera **(E)**, and species **(F)**. Bars represent the reads percentages found in metagenomic analyses.

To evaluate differences in phyla composition in the microbiota of feces samples obtained from patients and controls, we compare the relative abundances in both groups, represented by read percentages. The prevalent phyla in patients were Bacteroidetes [patient reads (*P*) = 47.97%; control reads (*C*) = 46.32%] and Firmicutes (*P* = 43.77%; *C* = 43.92%), the prevalent classes were Bacteroidia (*P* = 44.40%; *C* = 45.73%) and Clostridia (*P* = 35.31%; *C* = 34.39%), the prevalent orders were Bacteroidales (*P* = 44.41%; *C* = 45.73%) and Clostridiales (*P* = 35.31%; *C* = 34.39%), and the prevalent families were Bacteroidaceae (*P* = 21.09%; *C* = 27.19%), Ruminococcaceae (*P* = 19.05%; *C* = 17.99%), Prevotellaceae (*P* = 17.11%; *C* = 8.41%), and Lachnospiraceae (*P* = 11.00%; *C* = 12.43%) (Figures 2A–D). The prevalent genera in T2D patients were *Bacteroides* (*P* = 21.09%; *C* = 27.19%) and *Prevotella* (*P* = 14.07%; *C* = 7.03%). The prevalent species in the feces of T2D patients were *Prevotella copri* (*P* = 19%; *C* = 7%), *B. vulgatus* (*P* = 13%; *C* = 18%), *Bacteroides rodentium* (*P* = 9%; *C* = 13%), and *Bacteroides xylanisolvens* (*P* = 8%; *C* = 6%) (Figures 2E,F).

**Figure 3:**
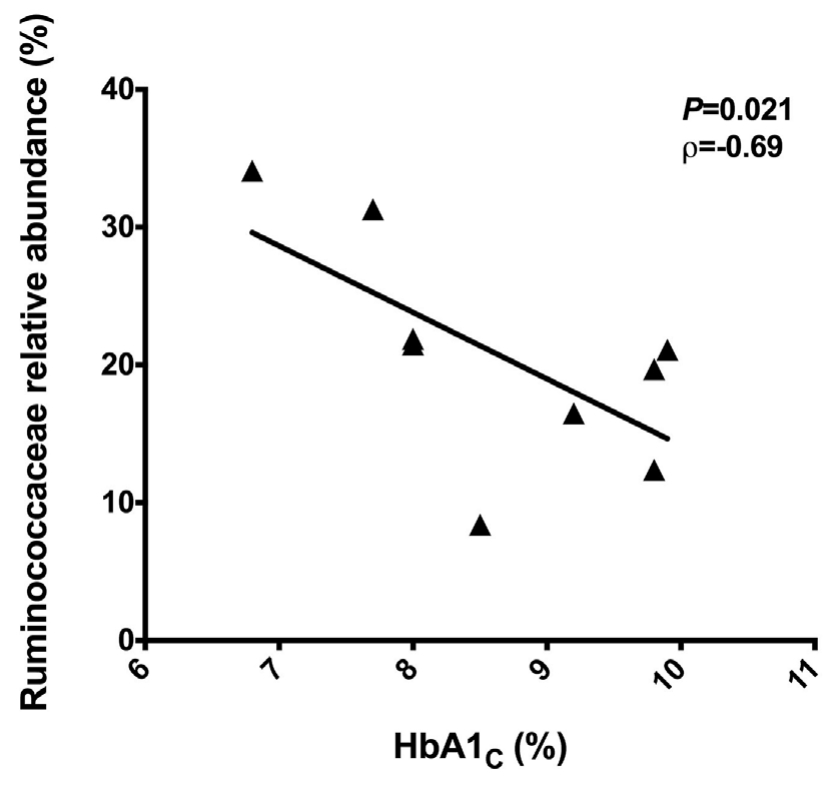
Negative correlation between the Ruminococcaceae reads with the HbA1_C_ percentages. Statistical analyses were performed using the Spearman’s test. Significance was set at *P* < 0.05.

To identify correlations between intestinal microbiota composition and clinical data, we examined the correlations among IMC, fasting blood glucose and HbA1_C_ with relative abundances of bacterial groups detected in the feces of T2D patients. There were no correlations among microbiota reads with IMC and fasting blood glucose. However, a negative correlation of the Ruminococcaceae reads with HbA1_C_ percentages (*P* = 0.021, ρ = −0.69) was observed (Figure 3).

### Proinflammatory IL-6 Is Increased in Plasma from T2D Patients

**Figure 4:**
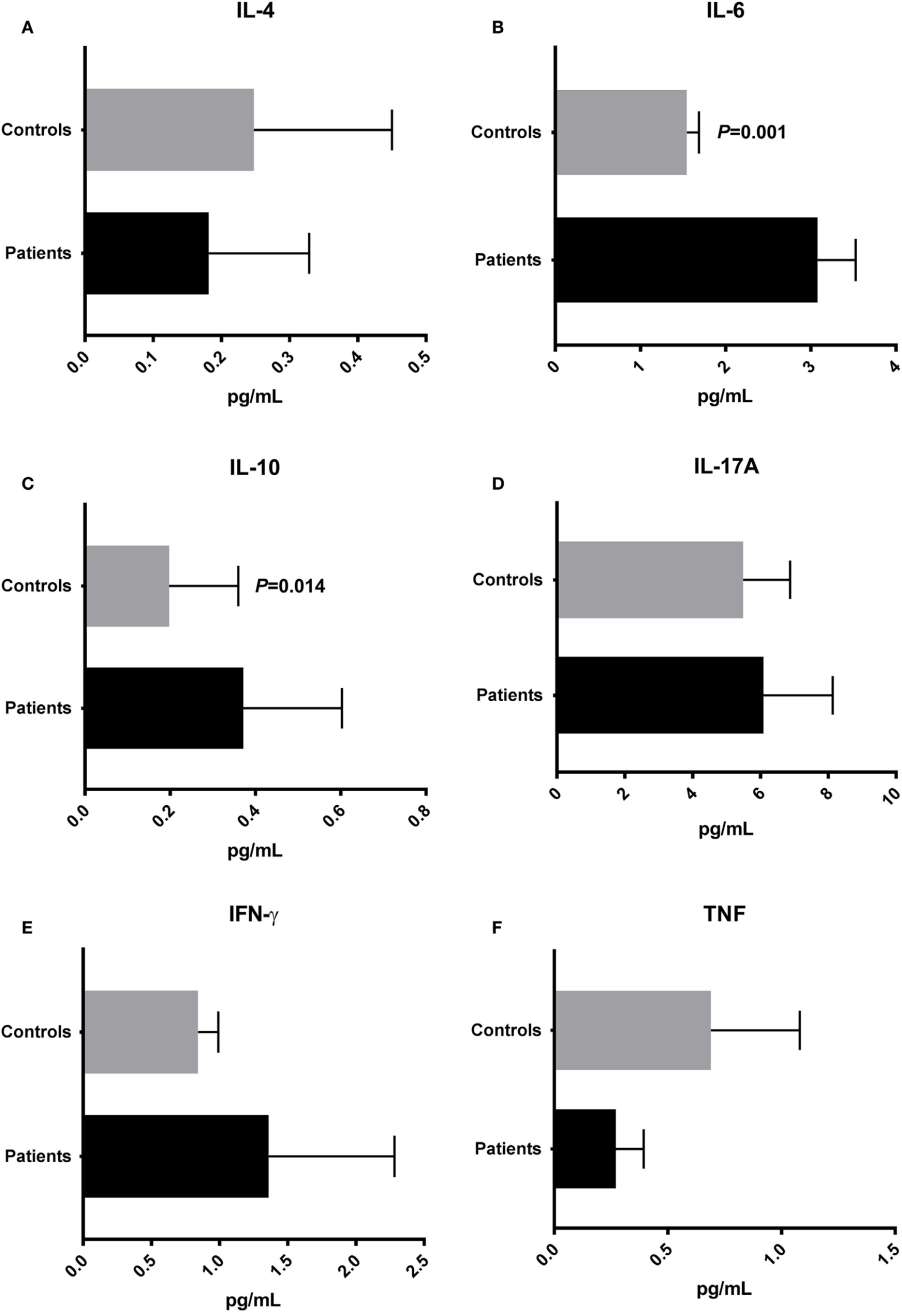
Cytokine profile in type 2 diabetes patients and control subjects. Plasma concentrations of IL-4 **(A)**, interleukin-6 (IL-6) **(B)**, IL-10 **(C)**, IL-17A **(D)**, interferon-gamma (IFN-γ) **(E)**, and tumor necrosis factor (TNF) **(F)**. Statistical analyses were performed by Mann–Whitney test. Significance was set at *P* < 0.05.

To evaluate the cytokine profile in plasma from T2D patients and controls, we quantified the plasma concentrations of IL-2, IL-4, IL-6, IL-10, IL-17A, IFN-γ, and TNF. IL-2 was undetectable in the vast majority of patient and control samples. IL-4 plasma concentrations, characteristic of Th2 responses, were similar in T2D (mean ± SEM: 0.181 ± 0.147 pg/mL) and controls (0.247 ± 0.203 pg/mL) (Figure 4A). The IL-6 concentration was significantly higher (*P* = 0.001) in patient plasma samples (3.081 ± 0.447 pg/mL) than in samples from the control group (1.547 ± 0.141 pg/mL) (Figure 4B). The concentration of IL-10, an anti-inflammatory cytokine, was increased (*P* = 0.014) in the plasma of patients (0.371 ± 0.231 pg/mL) compared with controls (0.197 ± 0.162 pg/mL) (Figure 4C). There were no significant differences (*P* > 0.05) in the plasma concentrations of proinflammatory cytokines IL-17A, IFN-γ, and TNF in patients (IL-17A: 6,089 ± 2,042 pg/mL; IFN-γ: 1.361 ± 0.921 pg/mL; TNF: 0.2714 ± 0,122 pg/mL) compared with controls (IL-17A: 5,491 ± 1,382 pg/mL; IFN-γ: 0.842 ± 0.149 pg/mL; TNF: 0.689 ± 0.391 pg/mL) (Figures 4D–F).

**Figure 5:**
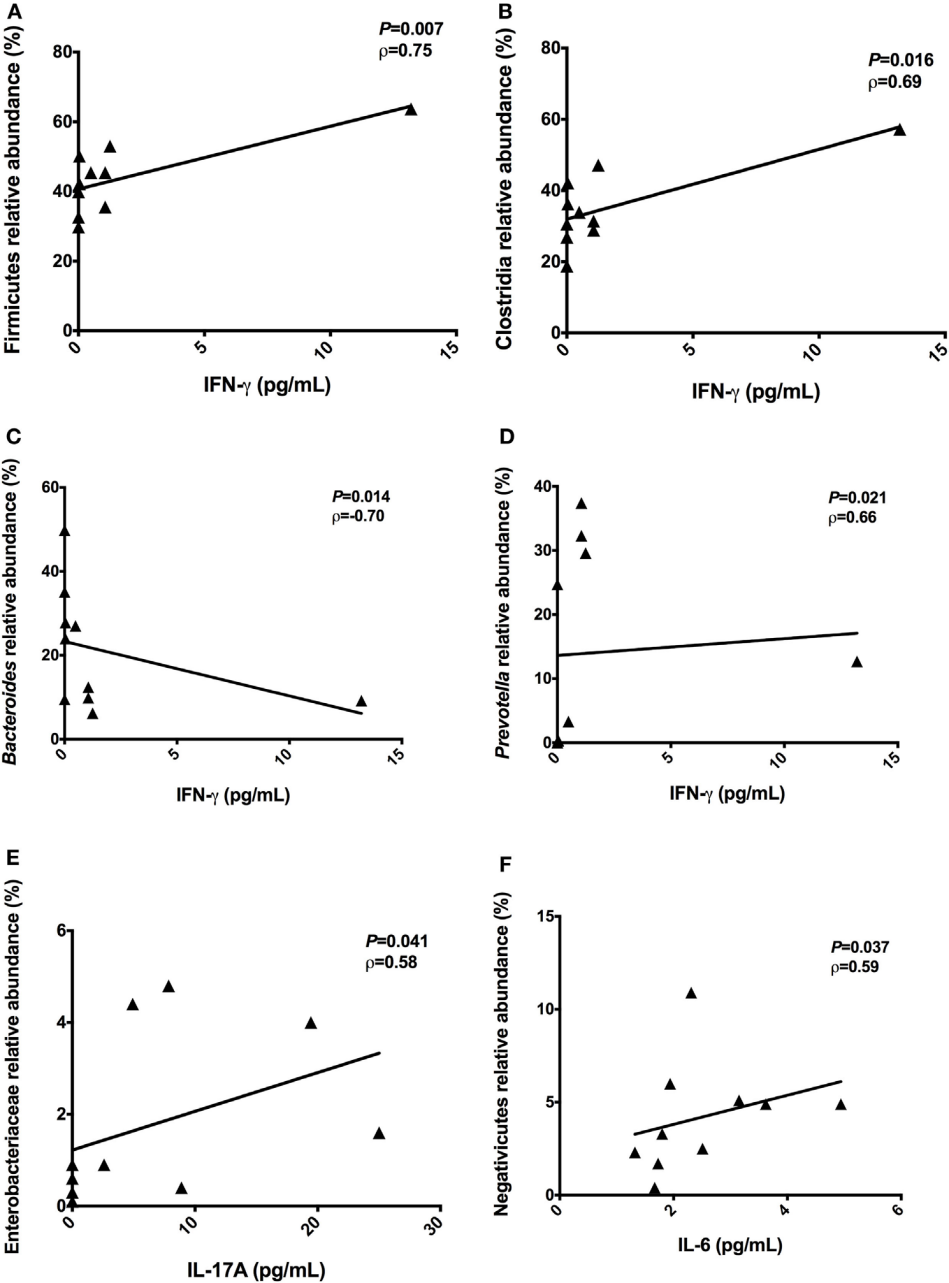
Correlations between the proinflammatory cytokines with relative abundances of bacterial taxa. Correlations found among interferon-gamma (IFN-γ) and Firmicutes phylum **(A)**, Clostridia class **(B)**, *Bacteroides* genus **(C)**, and *Prevotella* genus **(D)**. Positive correlation found between IL-17A with Enterobacteriaceae family **(E)**. Positive correlation found between interleukin-6 (IL-6) with Negativicutes class **(F)**. Statistical analyses were performed by Spearman’s test. Significance was set at *P* < 0.05.

To identify correlations between intestinal microbiota composition and cytokines, we examined correlations between systemic levels of cytokines and relative abundances of bacterial groups in the feces of T2D patients. Significant correlations among the proinflammatory cytokine IFN-γ and relative abundances of Firmicutes (*P* = 0.007, ρ = 0.75), Clostridia (*P* = 0.016, ρ = 0.69), *Bacteroides* (*P* = 0.014, ρ = −0.70), and *Prevotella* (*P* = 0.021, ρ = 0.66) were observed (Figures 5A–D). Plasma levels of IL-17A were positively correlated with the relative abundances of Enterobacteriaceae (*P* = 0.041, ρ = 0.58) (Figure 5E). Furthermore, a positive correlation between Negativicutes reads with IL-6 (*P* = 0.037, ρ = 0.59) was observed (Figure 5F).

### LPS Plasma Concentrations Were Different between Patients and Controls

**Figure 6:**
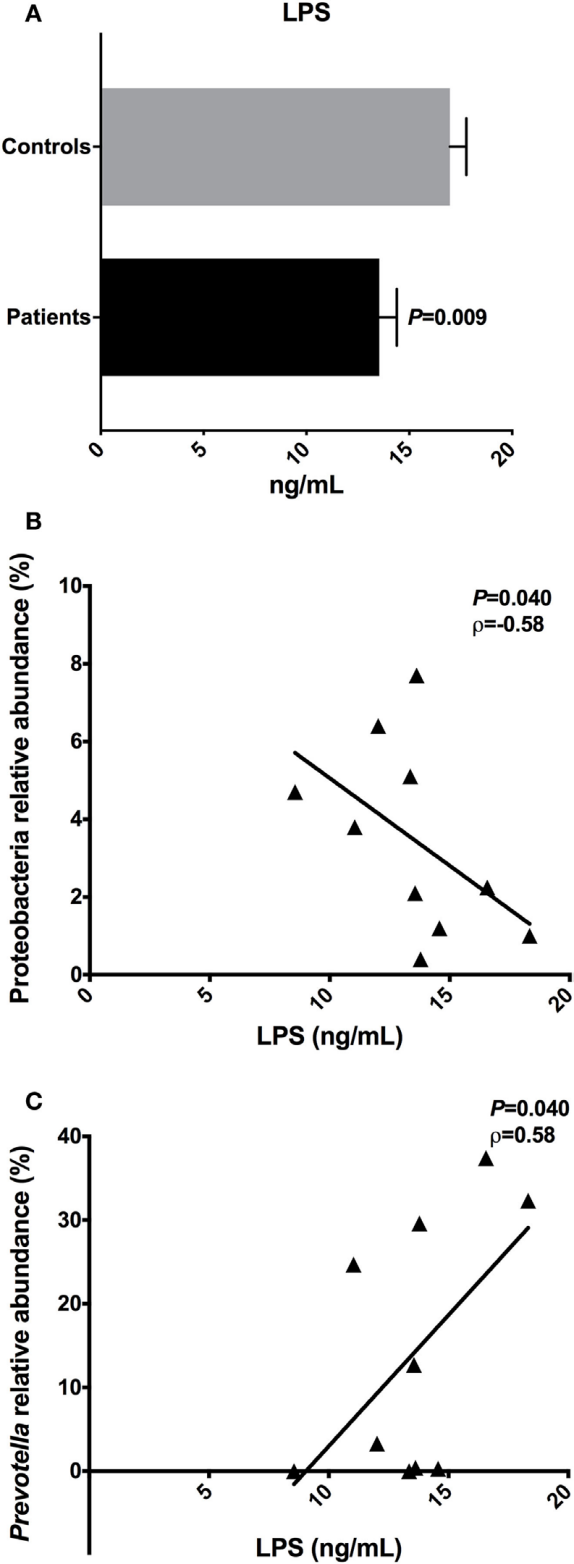
Lipopolysaccharide (LPS) concentrations and the correlations with relative abundances of bacterial taxa. Plasma levels of the LPS in type 2 diabetes patients and controls **(A)**. Negative correlation found between the LPS with Proteobacteria phylum **(B)**. Positive correlation found between the LPS with *Prevotella* genus **(C)**. Statistical analyses were performed using the Mann–Whitney and the Spearman’s test. Significance was set at *P* < 0.05.

To evaluate the metabolic endotoxemia in the plasma of T2D patients, we quantified LPS using an ELISA. We observed a significant difference (*P* = 0.009) in the LPS concentration in plasma from T2D patients (mean ± SEM: 13.54 ± 0.86 ng/mL) and controls (16.98 ± 0.79 ng/mL) (Figure 6A).

An inverse correlation between the LPS concentration in the plasma of patients and the relative abundance of Proteobacteria (*P* = 0.040, ρ = −0.58) was observed. Moreover, a positive correlation was observed between LPS and *Prevotella* reads (*P* = 0.041, ρ = 0.58) (Figures 6B,C). There was no correlation among LPS plasma levels detected in T2D patients, with concentrations of IL-4, IL-6, IL-10, IL-17A, IFN-γ, and TNF cytokines.

## Discussion

The human body is inhabited by several different microbial ecosystems that colonize the body’s mucous membranes (39, 40). Several studies have focused on the role of intestinal microbiota in health and disease, and factors that influence its dynamics, such as genetic and environmental factors (41, 42). A healthy intestinal microbiota is characterized by the presence of microorganisms that improve metabolism and confer resistance to infection and inflammation (43). Increasing evidence suggests that intestinal dysbiosis might be associated with the development of metabolic disorders, such as obesity and T2D (44–46). Here, we investigated the intestinal dysbiosis in Brazilian T2D patients and correlated these data with systemic inflammatory cytokines, LPS concentrations, and with clinical data.

The adult healthy gut microbiota is dominated by microorganisms belonging to the Firmicutes (Gram-positive) and Bacteroidetes (Gram-negative) phyla (39). Most bacteria in the adult human microbiota belong to the Firmicutes. and the prevalent species are *F. prausnitzii* and *Eubacterium rectale/Roseburia* spp. (47). These bacteria produce short-chain fatty acids, such as butyrate, which has anti-inflammatory effects (48). Butyrate inhibits NF-κB transcription factor signaling in intestinal epithelial cells and prevents the exposure of these cells to external factors, such as antigens derived from pathogenic microorganisms (47). The Bacteroidetes phylum is the second most populous in the human gut, with predominance of the *Bacteroides* and *Prevotella* genera (49). Studies have shown that *Prevotella* species predominantly activate TLR2 receptors and induce Th17 CD4 T cells polarization. The increased abundance of *Bacteroides* and *Prevotella* spp. is associated with gut inflammation, mainly mediated by proinflammatory Th17 cytokines. In addition, *Prevotella* spp. induce IL-8 and IL-6 secretion by epithelial cells, favoring Th17 responses and neutrophil recruitment (50). Thus, inflammation of the gut mucosa, mediated by *Prevotella* spp. promotes systemic dissemination of inflammatory mediators, increased intestinal permeability and translocation of bacterial products, which amplify and promote systemic inflammation (50).

In this study, we observed an intestinal dysbiosis in Brazilian T2D patients, with significant differences in the gut microbiota composition (beta-diversity) between patients and controls. Furthermore, we showed the prevalence of Gram-negative species in stool samples provided by patients, which is in agreement with previous studies (51, 52) and supports our hypothesis. The main Gram-negative species found in the present study were *P. copri, B. vulgatus, B. rodentium*, and *B. xylanisolvens*. The prevalence of Gram-negative bacteria suggests an increase in LPS levels, which can translocate through the intestinal barrier, and trigger systemic inflammation state and insulin resistance (51). Pedersen and colleagues showed that *P. copri* and *B. vulgatus* are associated with insulin resistance (52). In addition, studies in animal models have shown that *P. copri*, prevalent in our T2D patients, induce insulin resistance and glucose intolerance (52).

Alterations in the relative abundance of the Firmicutes and Bacteroidetes proportions have been reported in obesity and T2D (24, 53). Larsen and colleagues (2010) reported the diminished relative abundance of Firmicutes and Clostridia class in the diabetic group, while Bacteroidetes and Proteobacteria members were increased. These authors also observed a positive correlation between Bacteroidetes/Firmicutes and *Bacteroides-Prevotella*/*C. coccoides–E. rectale* ratios with plasma glucose concentrations (53). In the present study, we observed a negative correlation between Ruminococcaceae reads and HbA1_C_ percentages. Ruminococcaceae are a family of common anaerobes, Gram-positive gut microbes that break down complex carbohydrates, and these bacteria are most common in the intestine of individuals with carbohydrate-enriched diets (54).

Studies in T2D patients have reported decreased *R. intestinalis* and *F. prausnitzii*, both butyrate-producing bacteria (25). The prevalence of *Lactobacillus gasseri, Streptococcus mutans*, and *Akkermansia muciniphila* have also been reported in T2D patients (25, 53). In the present study, *R. intestinalis* and *F. prausnitzii*, butyrate-producing bacteria, were not observed. *A. muciniphila* was detected at a lower relative abundance in T2D patients and controls. A previous study demonstrated that this Gram-negative specie mediates the negative effect of IFN-γ on glucose tolerance in mice (55). Moreover, IFN-γ-deficient mice showed improved glucose metabolism, likely reflecting diminished adipose inflammation, hepatic gluconeogenesis, and increased insulin sensitivity (56–58). In the present study, we observed correlations between the proinflammatory IFN-γ with Gram-negatives *Bacteroides* and *Prevotella* species, also supporting our hypothesis.

One of the mechanisms proposed to explain the imbalance in the intestinal microbiota, the altered regulation of fat storage, and the development of metabolic diseases is the metabolic endotoxemia (59). Intestinal dysbiosis may trigger a state of chronic low-grade inflammation, making the host susceptible to systemic exposure to LPS (60). LPS is a potent inducer of innate immune responses and has been associated with the adiposity, insulin resistance, and *de novo* triglycerides synthesis (60). LPS binds to TLR4 and its coreceptor and triggers the inflammatory cascade, resulting in NF-κB activation and secretion of proinflammatory cytokines, such as TNF, IL-1, and IL-6, which influence glucose metabolism and inhibit the phosphorylation of insulin receptors (20, 21, 60).

In the present study, the proinflammatory IL-6 was increased in patients’ plasma and positively correlate with Negativicutes abundance. IL-6 is secreted from several cell types, such as macrophages, monocytes, dendritic cells, and T-cells (20). The stimuli for IL-6 synthesis include IL-1, TNF, and LPS (20). IL-6 influences the antigen-specific responses and inflammatory reactions and is one of the main mediators of acute phase reactions (1, 23). Jayashree and colleagues showed increased serum levels of LPS, TNF, and IL-6 in T2D patients when compared with controls (24). The authors also reported correlations among LPS with glucose concentrations, HbA1_C_ percentages, TNF, and IL-6 (24). In the present study, although there were no correlations between plasma inflammatory cytokines and LPS concentrations, the prevalence of Gram-negative species and the increased plasma IL-6 in patients could be associated with low-grade inflammation and insulin resistance.

Finally, we concluded that the *P. copri* and *B. vulgatus* species could represent an intestinal microbiota signature, associated with T2D development. Furthermore, the identification of these Gram-negative bacteria, and the detection of inflammatory markers, such as increased IL-6, could be used as diabetes predictive markers in overweight, obese, and genetically predisposed individuals to develop T2D.

## Ethics Statement

This study was carried out in accordance with the recommendations of Ethics Committee from Barretos Cancer Hospital with written informed consent from all subjects. All subjects gave written informed consent in accordance with the Declaration of Helsinki. The protocol was approved by the Barretos Cancer Hospital (Process number 903/2014).

## Author Contributions

AL participated in patient enrollment, sample collection, DNA extraction, cytokine and LPS quantification, data acquisition, and manuscript writing; MG and CS participated in patient enrollment and sample collection; NR and NS participated in V3/V4 amplification, library construction, and sequencing; JB provided support for control sample collection; EJ provided support to Illumina platform sequencing; JP was responsible for all clinical aspects involving T2D patients. WO and DP performed bioinformatics analyses; VM participated in sample collection, DNA, cytokine and LPS quantification, and data acquisition; GO participated in experimental conception, patient enrollment, sample collection, cytokine and LPS quantification, data interpretation, and manuscript writing and revision.

## Conflict of Interest Statement

The authors declare that the research was conducted in the absence of any commercial or financial relationships that could be construed as a potential conflict of interest.
